# Supplementary material for: Resolving the full spectrum of human genome variation using Linked-Reads
Source: Genome Res. 2019 Apr;29(4):635–45. doi: 10.1101/gr.234443.118 (PMC6442396; doi:10.1101/gr.234443.118)
Supplement: Supplemental Material [file supp_gr.234443.118_Supplemental_longranger-2.2.zip › longranger-2.2.tar.bz2!/lr2.2-public/lib/stan/cmdstan/stan/doc/api/html/structstan_1_1gm_1_1expression__grammar.html]

Stan: stan::gm::expression\_grammar< Iterator > Struct Template Reference


|  |  |
| --- | --- |
|  | Stan  2.5.0  probability, sampling & optimization |


- Main Page
- Related Pages
- Namespaces
- Classes
- Files

- Class List
- Class Index
- Class Hierarchy
- Class Members

All Classes Namespaces Files Functions Variables Typedefs Enumerations Enumerator Friends Macros Pages

- stan
- gm
- expression\_grammar

Public Member Functions |
Public Attributes |
List of all members

stan::gm::expression\_grammar< Iterator > Struct Template Reference

`#include <expression07_grammar.hpp>`

Inheritance diagram for stan::gm::expression\_grammar< Iterator >:

|  |  |
| --- | --- |
| Public Member Functions | |
|  | expression\_grammar (variable\_map &var\_map, std::stringstream &error\_msgs) |
|  | |

|  |  |
| --- | --- |
| Public Attributes | |
| variable\_map & | var\_map\_ |
|  | |
| std::stringstream & | error\_msgs\_ |
|  | |
| expression07\_grammar< Iterator > | expression07\_g |
|  | |
| boost::spirit::qi::rule  < Iterator, expression(var\_origin),   whitespace\_grammar< Iterator > > | expression\_r |
|  | |
| boost::spirit::qi::rule  < Iterator, expression(var\_origin),   whitespace\_grammar< Iterator > > | expression09\_r |
|  | |
| boost::spirit::qi::rule  < Iterator, expression(var\_origin),   whitespace\_grammar< Iterator > > | expression10\_r |
|  | |
| boost::spirit::qi::rule  < Iterator, expression(var\_origin),   whitespace\_grammar< Iterator > > | expression14\_r |
|  | |

## Detailed Description

### template<typename Iterator> struct stan::gm::expression\_grammar< Iterator >

Definition at line 23 of file expression07\_grammar.hpp.

## Constructor & Destructor Documentation

template<typename Iterator >

|  |  |  |  |
| --- | --- | --- | --- |
| stan::gm::expression\_grammar< Iterator >::expression\_grammar | ( | variable\_map & | *var\_map*, |
|  |  | std::stringstream & | *error\_msgs* |
|  | ) |  |  |

Definition at line 106 of file expression\_grammar\_def.hpp.

## Member Data Documentation

template<typename Iterator>

std::stringstream& stan::gm::expression\_grammar< Iterator >::error\_msgs\_

Definition at line 39 of file expression\_grammar.hpp.

template<typename Iterator>

expression07\_grammar<Iterator> stan::gm::expression\_grammar< Iterator >::expression07\_g

Definition at line 41 of file expression\_grammar.hpp.

template<typename Iterator>

boost::spirit::qi::rule<Iterator, expression(var\_origin), whitespace\_grammar<Iterator> > stan::gm::expression\_grammar< Iterator >::expression09\_r

Definition at line 52 of file expression\_grammar.hpp.

template<typename Iterator>

boost::spirit::qi::rule<Iterator, expression(var\_origin), whitespace\_grammar<Iterator> > stan::gm::expression\_grammar< Iterator >::expression10\_r

Definition at line 57 of file expression\_grammar.hpp.

template<typename Iterator>

boost::spirit::qi::rule<Iterator, expression(var\_origin), whitespace\_grammar<Iterator> > stan::gm::expression\_grammar< Iterator >::expression14\_r

Definition at line 62 of file expression\_grammar.hpp.

template<typename Iterator>

boost::spirit::qi::rule<Iterator, expression(var\_origin), whitespace\_grammar<Iterator> > stan::gm::expression\_grammar< Iterator >::expression\_r

Definition at line 47 of file expression\_grammar.hpp.

template<typename Iterator>

variable\_map& stan::gm::expression\_grammar< Iterator >::var\_map\_

Definition at line 37 of file expression\_grammar.hpp.

---

The documentation for this struct was generated from the following files:

- src/stan/gm/grammars/expression07\_grammar.hpp
- src/stan/gm/grammars/expression\_grammar.hpp
- src/stan/gm/grammars/expression\_grammar\_def.hpp


---

[ Stan Home Page ]

*© 2011–2014,
Stan Development Team.*
